# Effect of Ba:Ti Molar Ratio and Sintering Temperature on the Structural and Electrical Properties of BaTiO_3_-Type Solid Solutions, Synthesized by the Hydrothermal Method

**DOI:** 10.3390/ma18204797

**Published:** 2025-10-21

**Authors:** José Agustin Palmas Léon, Leandro Ramajo, Rodrigo Parra, Miguel Pérez Labra, Francisco Raúl Barrientos Hernández, Alejandro Cruz Ramírez, Vanessa Acosta Sanchez, Aislinn Michelle Teja Ruiz, Sayra Ordoñez Hernández

**Affiliations:** 1 Academic Area of Earth Sciences and Materials, Autonomous University of Hidalgo State, Road Pachuca-Tulancingo Km 4.5, Mineral de la Reforma 42184, Hidalgo, Mexico; pa373337@uaeh.edu.mx (J.A.P.L.); profe_3193@uaeh.edu.mx (F.R.B.H.); ac260546@uaeh.edu.mx (V.A.S.); aislinn_teja@uaeh.edu.mx (A.M.T.R.); 2 Instituto de Investigaciones en Ciencia y Tecnología de Materiales, National University of Mar del Plata, Av. Colón 10850, B7606 Mar del Plata, Buenos Aires B7600, Provincia de Buenos Aires, Argentina; lramajo@fi.mdp.edu.ar (L.R.); rparra@fi.mdp.edu.ar (R.P.); 3 Unidad Profesional Interdisciplinaria de Ingeniería Campus Hidalgo (UPIIH)—Instituto Politécnico Nacional, Carretera Pachuca-Actopan km 1-500, Distrito de Educación, Salud, Ciencia, Tecnología e Innovación, San Agustín Tlaxiaca 42162, Hidalgo, Mexico; alcruzr@ipn.mx; 4 Industrial Electromechanics Area, Technological University of Tulancingo, Tulancingo 43642, Hidalgo, Mexico; sayraoh@hotmail.com

**Keywords:** hydrothermal method, Ba:Ti molar ratio, sintering temperature, tetragonal ferroelectric BaTiO_3_, relative permittivity

## Abstract

The results of the effect of the three Ba:Ti molar ratios (MR) (1:1, 2:1, 4:1) and four sintering temperatures (1250, 1275, 1300, 1325 °C) on the structural and electrical properties of BaTiO_3_ (BT)-type ceramics synthesized by the hydrothermal method are shown. The BT phase formed was analyzed by x-ray diffraction (XRD), Raman spectroscopy (RS), dielectric and ferroelectric measurements and high-resolution scanning electron microscopy (HRSEM). For the samples synthesized using a Ba:Ti MR of 4:1 and at all sintering temperatures analyzed, XRD results confirmed the presence of the tetragonal ferroelectric phase, BT. In the same way, these results corroborated the results obtained by the RS technique. Dielectric properties measured at 100 kHz and 1 MHz over a temperature range of 30 °C–200 °C indicated a relative permittivity value of 4280 at 1 MHz and 4200 at 100 KHz at a Curie temperature of 110 °C in both cases for the sample synthesized at with a Ba:Ti MR ratio of 4:1 and sintered at 1300 °C. Ferroelectric measurements for the samples showed a best remnant polarization (Pr) of 3.5 µC/cm^2^ for the sample synthesized with a Ba:Ti MR ratio of 4:1 and sintered at 1325 °C. The HRSEM results showed grains composed of Ba, Ti, and O homogeneously distributed in the BT structure, and a trend of increasing average grain size with increasing sintering temperature was observed.

## 1. Introduction

BaTiO_3_ (BT) is a ceramic material with a perovskite-type structure, represented by the general formula ABX_3_, where A and B are cations and X the anion. These ions form octahedra, where cation A has 12 neighboring anions and cation B has 6 [1,2,3]. BT exhibits cubic symmetry (space group Pm3m), stable above 130 °C up to high temperatures (approximately 1460 °C), where the hexagonal phase is stable. Below 130 °C and up to 0 °C, it presents tetragonal symmetry (space group P4mm); between 0 °C and −88 °C, it becomes orthorhombic (space group Amm2); and at lower temperatures, it becomes rhombohedral [4,5]. BT is a ferroelectric material with outstanding dielectric, piezoelectric, and electro-optical properties, making it a key component in advanced electronic devices such as ceramic capacitors, sensors, actuators, non-volatile memories, power devices and in the manufacture of thermistors (materials with PTCR effect) [6]. It is well known that ferroelectricity, in the case of BT, originates from the low symmetry structure as follows: the Ti^4+^ ion moves with respect to the center of the oxygen octahedron, distorting the cubic lattice. This displacement occurs along certain preferred directions which are axes of order four in the tetragonal phase, of order two in the orthorhombic phase, and of order three in the rhombohedral phase. When the position of the Ti^4+^ ion changes, the interatomic bonding forces are altered, the covalence of the Ti-O bonds increases, and when the positive and negative charge centers do not coincide, an electric dipole appears [7]. It is clear then that the purity of BT phase is crucial, since traces of impurities or crystalline defects alter spontaneous polarization and generate charge dispersion centers, reducing the dielectric permittivity and increasing dielectric loss [8]. High purity ensures well-defined ferroelectric domains, improving stability and performance in electronic applications [7,8,9]. The purity of BT is, then obviously, related to the synthesis method used for the compound. BT perovskites can be synthesized using a variety of methods, including chemical, wet, thermal, and solid-state reaction routes [10,11,12,13,14,15]. All these methods have advantages and disadvantages that must be considered beforehand and on which the final characteristics of the ceramic obtained depend. Specifically hydrothermal method synthesis is a widely used technique for obtaining BT due to its numerous advantages over other conventional synthesis methods. Among its most notable benefits are: lower synthesis temperatures (generally between 100 and 250 °C) control over morphology and particle size, high purity and crystallinity, possibility of in situ doping, ecological and efficient process and scalability [16,17,18]. Together, these advantages make the hydrothermal method a highly attractive option for obtaining BT with optimal structural and functional properties, especially in electronic, dielectric, and piezoelectric applications. Nevertheless, to ensure a pure perovskite phase, it is essential to precisely control the Ba:Ti stoichiometric ratio and a suitable one heat treatment [19]. An unbalanced ratio or inadequate sintering can induce the formation of secondary phases such as BaTi_4_O_9_ or BaCO_3_ [20], which can negatively affect the perovskite structure and degrade the material’s dielectric properties, so optimizing these parameters is key to ensuring optimal functional performance.

For this reason, the present investigation focuses on experimentally determining the effect of Ba:Ti molar ratio and sintering temperature on the structural and electrical properties of BT-type solid solutions, synthesized by the hydrothermal method. This research aims to contribute to the understanding of the synthesis mechanism of BT with ferroelectric properties using the hydrothermal method, as well as its effects on its crystalline structure, charge mobility, and dielectric and ferroelectric properties.

## 2. Materials and Methods

BT-based ceramics were synthesized using the hydrothermal method to determine the effect of the Ba:Ti molar ratio and sintering temperature on its final structural and electrical and properties. The starting materials, titanium butoxide (Ti(OCH_2_CH_2_CH_2_CH_3_), CAS: 5593-70-4) and barium hydroxide (Ba(OH)_2_, CAS 12230-71-6) were mixed based on three molar ratios Ba:Ti (1:1, 2:1 and 4:1) to evaluate its effect on the purity of the obtained BT. The quantities in grams of reagents used for each Ba:Ti molar ratio are shown in Table 1. Each reagent mixture was placed inside a container of polytetrafluoroethylene (PTFE) and added to with deionized water until it reached a volume of 50 mL. Subsequently, 0.05 mol of sodium hydroxide (NaOH, Anedra CAS 1310-73-2) was added until an alkaline pH value was reached [21] (pH = 13). Additionally, to keep the particles dispersed within the container, 0.3 mmol of TRITON X-100 surfactant was added. Surfactant plays a crucial role as a particle-dispersing agent, as its presence reduces surface tension and prevents agglomeration during crystal growth. Surfactant molecules adsorb onto the surface of the nuclei and particles being formed, generating a steric or electrostatic barrier that prevents coalescence and promotes homogeneous distribution in the reaction medium. The PTFE container was hermetically sealed and placed inside a stainless-steel autoclave reactor, which was sealed and heated at 200 °C for 24 h inside an oven (INDEF 273 P) and later it was left to cool down to room temperature. The powders obtained were then filtered using 42 filter Ashless paper and washed thoroughly with an acetic acid (Cicarelli, San Lorenzo, Argentina, 99.5%, CAS: 64-19-7) solution (0.5 molar concentration in 50 mL of water distilled) to ensure the complete removal of remaining acetates and hydroxides. The obtained material was placed on a crystallizer and dried at 100 °C inside a drying oven. With the dry powders obtained, green pellets were formed using uniaxial pressure at 40 kgf in a 10 mm stainless steel die and were subsequently sintered inside a Carbolyte (38L787) furnace (Carbolite Gero, Hope, UK) equipped with a control Eurotherm 2404 at 1250 °C for 6 h in air with a heating and cooling rate of 4 °C per minute then smoothed using carbide abrasive paper, polished with a slurry of alumina, and cleaned in an ultrasonic bath. The structural evolution of the sintered pellets was monitored by X-ray diffraction analysis (XRD) (PANalytical X’Pert Cu Kα1 (1.54 Å) (PANalytical, Almelo, The Netherlands.) and a scanning angle 2θ of 20 to 80° with a step ~0.02° 2θ) and the molar ratio with which the purest BT phase was obtained was then selected to produce four green pellets using the same pressure and dimensions mentioned above. The obtained pellets were sintered in air at four temperatures: 1250, 1275, 1300 and 1325 °C using a heating and cooling ramp of 4 °/min for 4 h inside a Carbolyte (38L787) furnace equipped with a Eurotherm 2404 controller. The composition of the sintered pellets were analyzed using a high-resolution scanning electron microscope (HRSEM, JEOL 6701 F, JEOL Ltd., Tokyo, Japan) through of point energy dispersive microanalysis (EDS) (AV = 15 kV, WD = 9.9, PC = 11 and DT = 15 s). The structural evolution was then carried out by X-ray diffraction analysis (XRD) (PANalytical X’Pert Cu Kα1 (1.54 Å) and a scanning angle 2θ of 20 to 80° with a step ~0.02° 2θ). Additionally, to achieve a better analysis of the peak splitting area corresponding to the (002) plane of the tetragonal phase of the fabricated BT, acquisitions were performed in the range 2θ = 43–47°, with a step value of 0.01° and an acquisition time of 5 s.

The analysis of the results showed that the lattice parameters and crystallite size in the BT samples sintered at different temperatures using the Scherrer equation [22]:

(1) $$D=\frac{K*\lambda }{\beta *\cos\theta }$$

where *D* is the average crystallite size (Å), *K* is the shape constant (≈0.9), λ is the wavelength of the X-ray source (Cu Kα1 = 1.54 Å), *β* is the peak width at half height (FWHM, rad), and θ is the diffraction angle (rad). Raman analyses were performed at room temperature using a Renishaw InVia Raman spectrometer (Renishaw, Wotton-under-Edge, Gloucestershire, UK) coupled to a Leica DMLM microscope (Leica, Wetzlar, Germany) over the range of 100–1100 cm^−1^ wavenumbers. The spectra were acquired with the 50xN Plan EPI (0.50 aperture). To focus on and search for points of interest, the microscope implements a motorized stage (XYZ). A 785 nm diode pumped solid state Nd:YAG (neodymium-doped yttrium aluminum garnet) laser was used in all measurements with 50 mW nominal power at source. Normally, 1–5 scans, each lasting 120 s, are accumulated to achieve a suitable signal-to-noise enhancement at an operating spectral resolution of ≤1 cm^−1^. Dielectric properties were performed using an LCR HiTester and an impedance meter HP 4284A LCR meter in the range of 100 Hz–1 MHz over a temperature range of 25–200 °C for which, the opposite faces of the pellets were painted with silver-platinum paint, and a thin strip of platinum was placed on each side to act as electrodes. For the relative permittivity calculations, the following equation was used [23]:

(2) $$\varepsilon^{\prime }=\frac{C*d}{\varepsilon_{0}*A}$$

where *ε*′ is the relative permittivity of the ceramic (F/m), *C* is the capacitance (F), *d* is the thickness of the sample (mm), *A* is the area of the electrode (mm^2^) and *ε*_0_ is the absolute permittivity of the vacuum (8.854 × 10^−12^ F·m^−1^).

For the determination of ferroelectric properties, the pellets coated on their opposite sides with silver-platinum paste were placed in a silicone oil bath at room temperature and a sinusoidal wave electric field at a frequency of 50 Hz was applied by means of a Sawyer–Tower bridge, coupled to an HP 4284A impedance analyzer in the frequency range of 20 Hz–1 MHz using a Novocontrol BDS1200 cell (Novocontrol Technologies GmbH & Co. KG, located in Montabaur, Germany). The particle size distribution and morphology were characterized by high-resolution scanning electron microscopy (HRSEM JEOL 6701F, JEOL Ltd., Tokyo, Japan) measuring each grain observed in 5 micrographs for each pellet and averaging these values employing ImageJ software 1.54p [24]. Finally, the determination of apparent density (ρ) and apparent porosity (P) of the sintered pellets was carried out by the Archimedes method using a high-sensitivity Sartorius hydrostatic balance using the following equations [25,26]:

(3) $$\rho =\frac{w_{dry}}{W_{sat}-W_{sub}}\rho_{f}$$

(4) $$P=\frac{W_{sat}-w_{dry}}{W_{sat}-W_{sub}}*100$$

where ρ is the apparent density (g/cm^2^), *W_dry_* is the weight of the dry sample (g), *W_sat_* is the weight of the sample saturated in air (g), *W_sub_* is the weight of the sample saturated in water (g) and *P* is the apparent porosity (%).

## 3. Results

### 3.1. X-Ray Diffraction

#### 3.1.1. Effect of the Ba:Ti Molar Ratio on the Synthesis of BT

**Figure 1:**
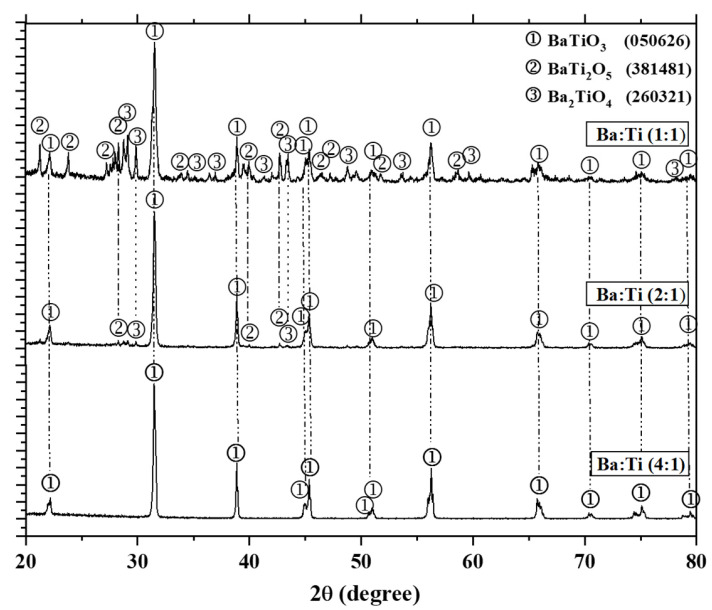
XRD patterns of the powders samples synthesized with different Ba:Ti molar ratios and sintered at 1250 °C for 4 h in air atmosphere.

The XRD patterns obtained for the BT-based ceramics synthesized at different Ba:Ti molar ratios and sintered at 1250 °C for 4 h in air atmosphere are shown in Figure 1. For the samples where the Ba:Ti molar ratio was 1:1 and 2:1, a mixture of the secondary phases BaTi_2_O_5_ (JCPDS 381481) and Ba_2_TiO_4_ (JCPDS 260321) with the tetragonal ferroelectric phase BT (JCPDS 020626) was identified. The Ba_2_TiO_4_ phase (barium orthotitanate) is a ceramic material belonging to the BaO-TiO_2_ system and exhibits an orthorhombic crystal structure and is characterized by its high thermal and chemical stability, as well as its low dielectric constant compared to other barium titanates such as BT and its presence can negatively affect the ferroelectric and dielectric properties of materials designed for electronic applications [20,27,28]. The formation of the secondary phases BaTi_2_O_5_ and Ba_2_TiO_4_ is related to an asymmetric kinetics between the Ba^2+^ and (Ti(OH)_6_)^−2^ ions, causing a low local composition equilibrium [29]. Another cause for the formation of these secondary phases is attributed to the excess of titanium in solutions with alkaline pH [13], because barium has low solubility and therefore a lower reaction with Ti ions [27]. On the other hand, it is also observed in Figure 1 that in the XRD pattern obtained for the sample synthesized using a Ba:Ti molar ratio of 4:1, only the tetragonal ferroelectric phase BT (JCPDS 020626) was identified at the positions 2θ ≈ 22, 31, 38, 45, main peaks, which indicated a maximum purity of the BT phase. The splitting of the (002) and (200) planes at the peak near 45° in the XRD pattern (particularly in the region between 44° and 46° 2θ with Cu Kα radiation) is a key feature that reveals important structural information about this material [12]. This phenomenon is directly associated with the tetragonal crystal structure of BT at room temperature [30]. At this stage, due to tetragonal distortion, the (002) and (200) planes are no longer equivalent, resulting in the appearance of two separate peaks instead of one. However, if BT adopts a cubic structure, these planes become equivalent and the diffractogram shows a single peak in that region, since there is no distortion to differentiate them. Therefore, the presence of splitting is a direct indicator of the tetragonal phase, which is closely related to the ferroelectric property of BT [31]. Its analysis is essential to confirm the crystalline phase, evaluate the structural purity of the material and correlate the microstructure with its functional properties.

#### 3.1.2. Effect of Sintering Temperature

**Figure 2:**
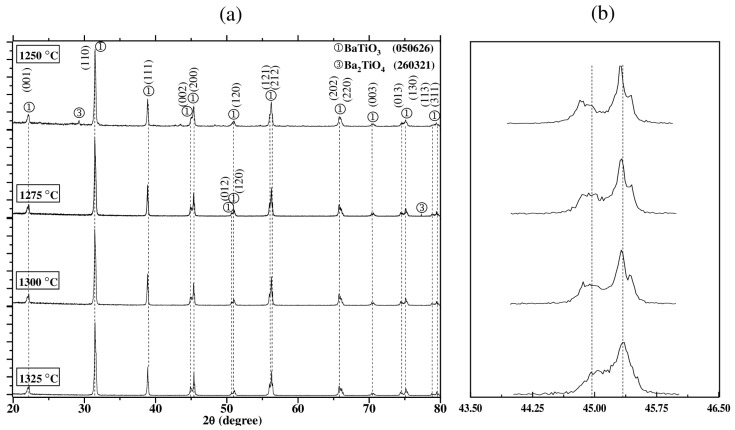
(**a**) XRD diffraction patterns of the samples sintered in air at 1250, 1275, 1300 and 1325 °C for 4 h and using a heating and cooling ramp of 4 °C/min. (Ba:Ti = 4:1). (**b**) Zoom at 2θ ≈ 43.5–56.5°.

The sintering temperature of BT is a key factor in determining its microstructure, crystalline phase, and functional properties [16,17,18,19,20]. An appropriate temperature allows for greater densification and controlled grain growth, which directly influences its dielectric and ferroelectric properties [20,28]. On the other hand, poor sintering temperature can generate secondary phases or structural defects that negatively affect its performance in electronic applications [7,8,9]. In this study, according to the results shown in Figure 1, the effect of the sintering temperature was evaluated by analyzing four samples synthesized using a Ba:Ti molar ratio of 4:1, testing four different sintering temperatures: 1250, 1275, 1300 and 1325 °C and using a heating and cooling ramp of 4 °C/min. The XRD patterns obtained of each sintered sample are shown in Figure 2a. It was observed in the results corresponding to the samples sintered at 1250 °C and 1275 °C that a mixture of barium orthotitanate (Ba_2_TiO_4_) (JCPDS 260321) in the positions 2θ ≈ 29.20, 77° and BT (JCPDS 050626) phases were identified. In contrast, for the samples sintered at 1300 and 1325 °C, only the presence of the ferroelectric tetragonal phase BT (JCPDS 050626) was identified. Again, the stability of the tetragonal phase was corroborated due to the presence of the splitting of the (002) and (200) planes at the peak close to 45° [20], indicating a correct evolution of the orthorhombic phase (Amm2) to the tetragonal phase (P4mm) [28].

In Figure 2b, the evolution of the splitting of the (002) and (200) planes is shown and a slight shift to the right as the sintering temperature was increased was observed. This phenomenon was attributed to the lattice distortion that emerges as the unit cell lattice parameters expand; such expansion induces stress and internal disorder consistent with observations in advanced ceramics [32].

The structural evolution of the BT phase was studied through changes in its lattice parameters (*a* and *c*) at each sintering temperature. The computed lattice parameters, the tetragonality ratio (*c*/*a*) and the crystallite size were calculated using Equation (1) and are shown in Table 2. It is well known that the *a*/*c* parameter is a direct indicator of the displacement of the Ti^4+^ ion within the TiO_6_ octahedron, which generates a dipole moment responsible for ferroelectricity [33]. A *c*/*a* ratio greater than 1 indicates tetragonal distortion, indicating strong spontaneous polarization. On the other hand, the crystallite size of BT refers to the size of the coherently ordered regions within each particle or grain and can significantly influence its structural and functional properties [19]. The tetragonality ratio (*c*/*a*) calculated in all samples was measured >1, which indicated the tetragonality of the analyzed systems [29]. Similarly, the tetragonality parameter showed a maximum value of 1.0113 when the sintering temperature employed was 1300 °C, which indicated that this sample could present the best ferroelectric properties. This result was consistent with what was shown in Figure 2a, since the sample sintered at 1300 °C did not show secondary phases. The tetragonality ratio (*c*/*a*) in BT is directly linked to its primary crystalline phase and the structural quality of the material. When secondary phases (such as Ba_2_TiO_4_, or unreacted TiO_2_) appear, this ratio may be altered or decreased, as these phases do not present the same crystalline structure nor contribute to spontaneous polarization [34]. From the results shown in Table 2, a growth in crystallite size of the peak located at 2θ ≈ 45° was observed as the sintering temperature was increased from 1250 to 1325 °C with a value of 3540.87 Å at 1300 °C indicating a greater structural order within the material, which is usually associated with better crystallization and greater stability of the tetragonal phase [35].

### 3.2. Raman Spectroscopy

**Figure 3:**
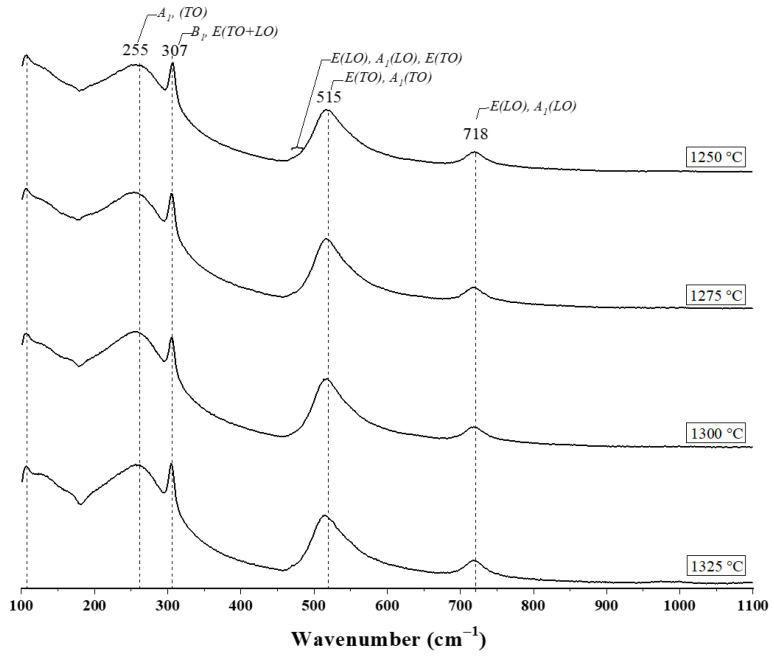
Raman spectra of BT samples synthesized in air by the hydrothermal method using a Ba:Ti molar ratio = 4:1 and sintered in air at 1250, 1275, 1300 and 1325 °C and a heating and cooling ramp of 4 °C/min.

Raman spectroscopy is a fundamental technique for the structural characterization of BT ceramics as it allows the identification of its crystalline phases and the non-destructive detection of structural defects or local distortions [36]. The presence and position of certain active modes in the Raman spectrum indicate whether the BT retains the ferroelectric tetragonal phase, which is key to its technological applications. Figure 3 shows the Raman spectra of BT samples synthesized in air by the hydrothermal method using a Ba:Ti molar ratio = 4:1 and sintered at 1250, 1275, 1300 and 1325 °C and a heating and cooling ramp of 4 °C/min. It shows the characteristic depolarized scattering profiles for single and polycrystalline BT [36,37,38]. The band located in 307 cm^−1^ (*B*_1_, *E*(TO + LO)) it is related to the antisymmetric B1g vibration mode of the cations at the B site It is related to the antisymmetric B1g vibration mode of the cations at the B site (Ti^4+^) and the O^2−^ anion [36]. On the other hand, the band located at 515 cm^−1^ is attributed to the mode (*E*(TO), *A*_1_(TO)) and is similarly associated with the vibration of the cations at site B (Ti^4+^) and the O^2−^ anion [36]. The difference between the bands at 307 and 515 cm^−1^ is that the band at 307 presents anti-symmetry, while that of 515 is symmetrical and the vibration is carried out transversely [36]. The band at 255 cm^−1^ corresponds to the A_1_(TO) mode of the cations at the B site (Ti^4+^) and the O^2−^ anion [37]—this vibration occurs when the symmetry of the octahedron is lost. The bands at 105 and 718 cm^−1^, respectively, are characteristic of the tetragonal structure of BT [38,39,40]. Table 3 summarizes the vibration modes of BT samples sintered at different temperatures.

### 3.3. High-Resolution Scanning Electron Microscopy (HRSEM)

**Figure 4:**
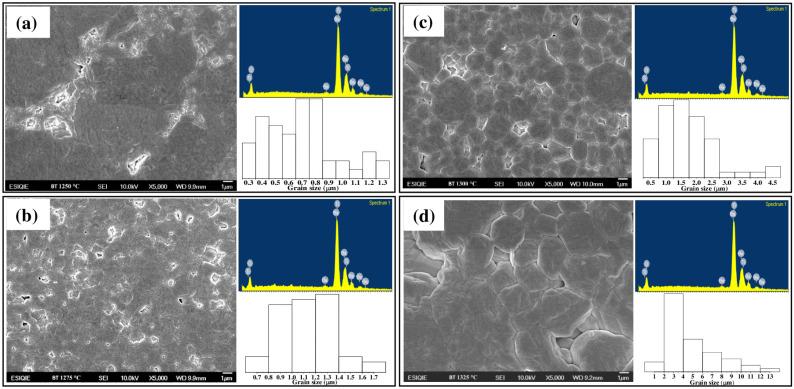
HRSEM micrographs, point energy dispersive microanalysis (EDS) and grain size histograms of BT ceramics synthesized in air atmosphere with a Ba:Ti molar ratio of 4:1 and sintered at: (**a**) 1250 °C, (**b**) 1275 °C, (**c**) 1300 °C, and (**d**) 1325 °C for 4 h using a heating and cooling ramp of 4 °C/min.

Figure 4 displays the HRSEM micrographs, the point EDS microanalysis and grain size histograms of BT ceramics synthesized in air atmosphere with a Ba:Ti molar ratio of 4:1 and sintered at 1250, 1275, 1300, and 1325 °C for 4 h using a heating and cooling ramp of 4 °C/min. In all samples, the point energy dispersive microanalysis (EDS) performed using an acceleration voltage of 15 kV, working distance = 9.9, probe current = 11 and date time = 15 s., revealed that the grains were composed of barium, titanium, and oxygen elements homogeneously distributed near their surfaces. The grain size distributions determined by measuring each grain observed in five micrographs for each pellet and averaging these values using ImageJ software are included [24]. In Figure 4a, grains with an average size of ≈ 0.73 µm were observed. This behavior was also observed in the sample sintered at 1275 °C (Figure 4b), with an increase in the average grain size to >1 µm. In general, the particles exhibited a faceted morphology, a characteristic commonly observed in undoped BT ceramics [13]. In Figure 4c,d an increase in the average grain size to 1.94 and 4.67 µm, respectively, was determined. The grains were characterized by faceted shapes with sub-rounded edges. The increase in grain size shows that increasing sintering temperature in BT can promote atomic diffusion and thus accelerate grain growth [41].

**Figure 5:**
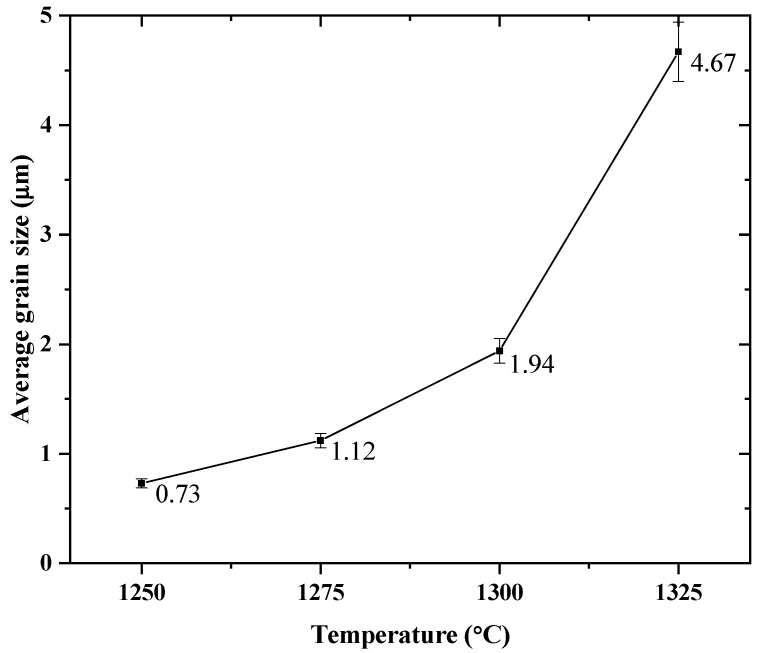
Evolution of the grain size of the BT samples as a function of the sintering temperature.

Figure 5 graphically shows the evolution in grain size in relation to the sintering temperature applied to each sample. It has been reported [42] that grain growth of BT can be beneficial up to a certain size, since a denser microstructure with uniform grains improves the dielectric and ferroelectric properties. However, if the temperature is excessive or the sintering time is too long, abnormal grain growth may occur, which could generate a non-uniform size distribution, decrease the grain boundary density and may adversely affect the functional properties [42,43].

#### Density and Apparent Porosity

**Figure 6:**
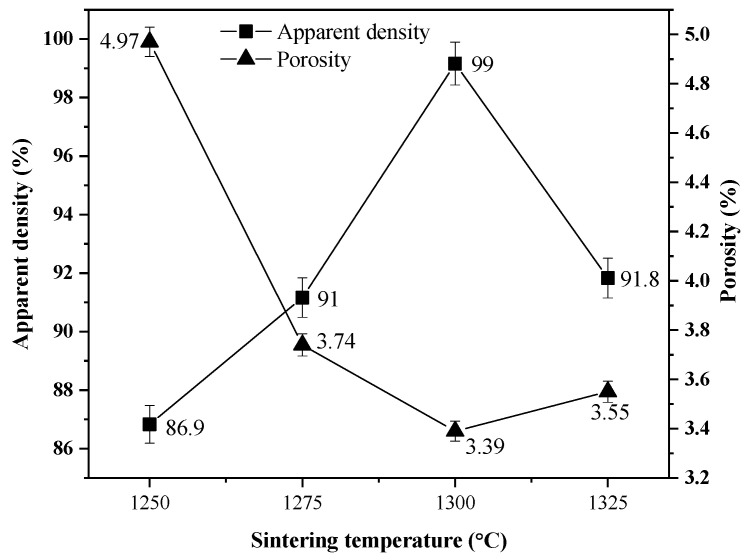
Density and apparent porosity of BT ceramics synthesized with a Ba:Ti molar ratio of 4:1 and sintered in air atmosphere at 1250, 1275, 1300, and 1325 °C for 4 h using a heating and cooling ramp of 4 °C/min.

The results of density and apparent porosity of BT ceramics synthesized in air atmosphere with a Ba:Ti molar ratio of 4:1 and sintered at 1250, 1275, 1300, and 1325 °C for 4 h using a heating and cooling ramp of 4 °C/min calculated with Equations (3) and (4) are shown in Figure 6. A decrease in porosity could be observed with increasing sintering temperature. The temperature of 1300 °C presented the lowest amount of porosity and the highest percentage of apparent density: 99.16% (5.95 g/cm^3^), an optimal value in relation to the theoretical density, which indicated ideal sintering conditions. Excessive grain size increases in BT can reduce the density of the ceramic because during accelerated growth the grains tend to join unevenly, generating residual pores that become trapped in the microstructure [44].

Based on the previously shown results of XRD, Raman spectroscopy and HRSEM, it can be established that the temperature of 1300 °C presented the best microstructural (theoretical density of 99.16%) and crystalline (stability of the tetragonal phase at room temperature, *c*/*a* > 1) characteristics to evaluate its electrical properties.

### 3.4. Dielectric Properties

**Figure 7:**
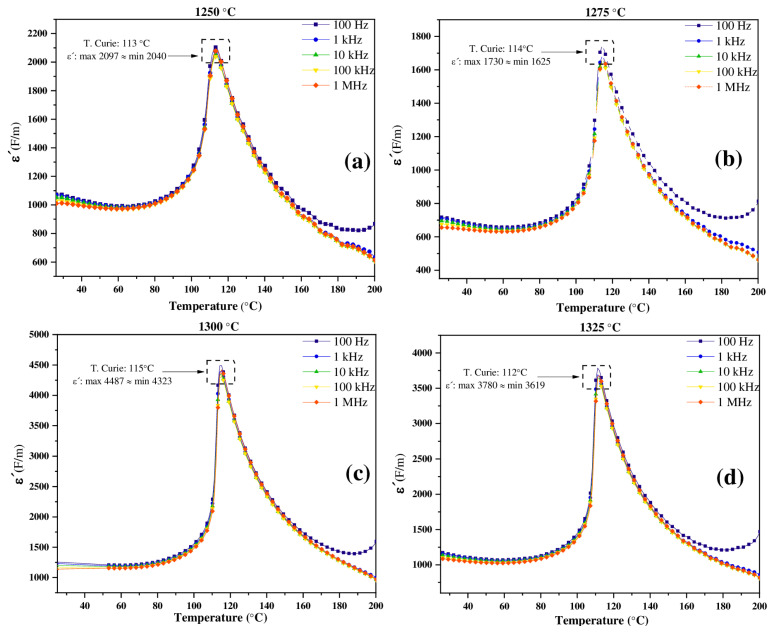
Relative permittivity (*ε*′) measured from 100 Hz to 1 MHz for BT capacitor devices synthesized by the hydrothermal method using a Ba:Ti molar ratio of 4:1, sintered in air at: (**a**) 1250 °C, (**b**) 1275 °C, (**c**) 1300 °C and (**d**) 1325 °C and using a heating and cooling ramp of 4 °C/min.

The relative permittivity (*ε*′) graphs measured from room temperature to 200 °C and evaluated in the range of 100 Hz–1 MHz for BT capacitor devices synthesized by the hydrothermal method using a Ba:Ti molar ratio of 4:1, sintered in air atmosphere at 1250, 1275, 1300 and 1325 °C using a heating and cooling ramp of 4 °C/min are shown in Figure 7. The *ε*′ plots present, for all the samples evaluated, a single phase transition, assigned to the tetragonal to cubic paraelectric phase transition [45] at a Curie temperature (TC) of 113, 114, 115, and 112 °C for the samples sintered in air atmosphere at 1250, 1275, 1300 and 1325 °C using a heating and cooling ramp of 4 °C/min, respectively. This analysis also reveals the dependence of the relative permittivity on the microstructure of the samples, the sample sintered at 1300 °C showed the highest permittivity values (*ε*′max = 4487, *ε*′min = 4323). This behavior was attributed to the ability of the material dipoles to align with the applied external electric field in samples with high tetragonality, even at the high applied frequencies [46,47,48]. In BT samples, *ε*′ is strongly related to the tetragonality ratio (*c*/*a*), since the latter is a direct indicator of the structural distortion and, therefore, the spontaneous polarization of the material [49]. The above results are consistent with those obtained in Figure 2, Table 2 and Figure 3.

Dielectric loss (tan δ) in BT refers to the fraction of electrical energy that the material dissipates as heat when subjected to an alternating electric field. A low dielectric loss value is desirable for applications such as capacitors, as it implies that the material can store and release energy efficiently without dissipating it excessively as heat [50]. Tang δ, in this paper, was calculated for the results obtained at the frequencies of 100 kHz and 1 MHz for samples sintered at 1250, 1275, 1300 and 1325 °C, because these frequencies presented the highest values of *ε*′.

**Figure 8:**
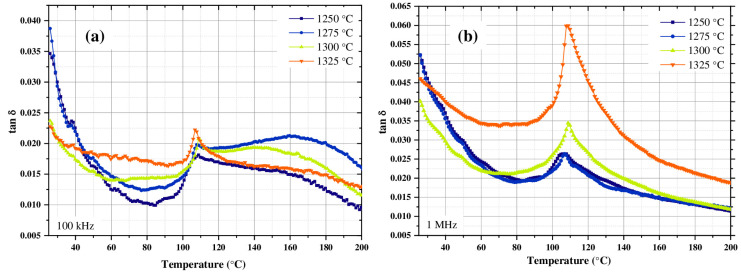
tan δ measured at (**a**) 100 kHz, (**b**) 1 MHz for BT capacitor devices synthesized by the hydrothermal method using a Ba:Ti molar ratio of 4:1, sintered at 1250, 1275, 1300 and 1325 °C in air atmosphere and using a heating and cooling ramp of 4 °C/min.

In Figure 8a,b, it can be noted that the BT sample sintered at 1325 °C, presented the highest value of tan δ. This was attributed to the relaxation mechanisms of the dipole charges, which is explained by the decrease in the observed density (Figure 6). Dielectric loss tends to increase as the density of the material decreases, and this relationship is explained by the resulting microstructure [51]. For their part, the samples sintered at 1250, 1275 and 1300 °C presented losses that were closely similar to each other. This is explained because with the use of sintering temperatures close to the ideal (sample sintered at 1300 °C) it allows for high density (Figure 6), good bonding between grains and a homogeneous microstructure, which reduces charge barriers and leakage currents, producing low dielectric losses [52]. As shown in Table 2, the sample sintered at 1300 °C showed greater lattice distortion—this facilitates the mobility of the dipoles causing the release of energy associated with the phase transition (tan δ peak at 110 °C) [53].

On the contrary, high sintering temperatures (sample sintered at 1325 °C) generated abnormal grain growth (Figure 4d) and pore entrapment may occur within the grains, as well as volatilization of BaO, which introduces defects and again increases the dielectric loss [54]. It is also observed in Figure 8b that the values of tan δ measured at 1 MHz were significantly higher than those obtained at 100 kHz (Figure 8a). This was attributed to the fact that after a certain frequency threshold, the increase in this usually causes an increase in tan δ due to the dynamics of the polarization mechanisms, which is explained by the fact that the dipoles cannot follow the electric field as quickly, causing an increasing phase shift between polarization and applied field, increasing energy dissipation. Furthermore, certain polarization mechanisms “freeze” as the frequency increases, generating relaxation processes that increase losses [55]. On the contrary, at low frequencies, all forms of polarization present in the material can follow the oscillations of the electric field, resulting in moderate losses [55]. In the kHz–MHz range, the increase in frequency is associated with higher dipolar and interfacial relaxation losses [53,54,55].

**Figure 9:**
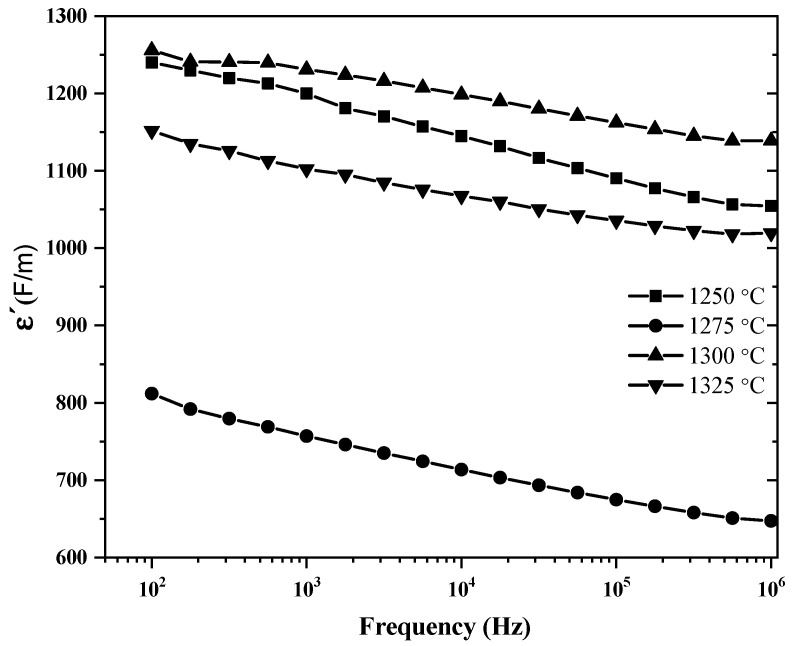
Representation of the relative permittivity values at room temperature as a function of the applied frequency of BT capacitor samples sintered at different temperatures in air atmosphere for 4 h.

Figure 9 shows graphically the representation of the relative permittivity values as a function of the applied frequency, at room temperature for the samples sintered at different temperatures. The BT sample sintered at 1300 °C showed the highest permittivity (≈1260) at low frequencies. In general, it was observed that all the samples evaluated presented instantaneous polarization values in different magnitudes, attributed to the structural asymmetry induced by ionic displacement, reinforced by the ferroelectric nature of BT [53].

### 3.5. Ferroelectric Analysis

**Figure 10:**
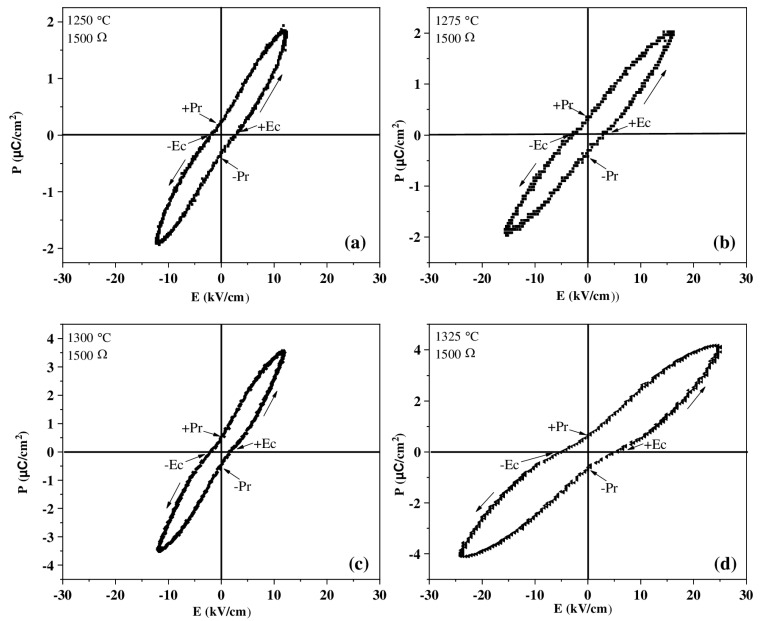
P–E loops for BT capacitor devices sintered at different temperatures employing a resistance of 1500 Ω. (**a**) Sintered sample at 1250 °C; (**b**) sintered sample at 1275 °C; (**c**) sintered sample at 1300 °C; (**d**) sintered sample at 1325 °C.

Figure 10 shows the evaluation of BT ceramics sintered at different temperatures in their ferroelectric behavior employing a resistance of 1500 Ω. The P–E loops, who presented a fairly smooth shape characteristic of a soft ferroelectric, shown that the saturation polarization (*Ps*) and the remnant polarization (*Pr*) was increased with increasing sintering temperature, indicating a larger remaining dipole moment, related to the grain size observed in HRSEM (Figure 4) and the density values obtained (Figure 6) [54]. On the other hand, at low fields, all samples behaved essentially as linear paraelectric. Table 4 shows the remanent polarization (*Pr*) values of BT capacitor devices as a function of their sintering temperature In BaTiO_3_, grain growth during the sintering process has a direct influence on its dielectric and ferroelectric properties. As grain size increases, the proportion of grain boundary regions, which act as polarization barriers and charge dispersion centers, decreases [42]. This favors greater mobility and alignment of ferroelectric domains, increasing the dielectric constant and remanent polarization. Conversely, in materials with very fine grains, the high density of boundaries restricts the movement of domain walls, reducing polarization capacity and, consequently, the ferroelectric response [27,42]. However, excessive grain growth can lead to heterogeneity and facilitate the formation of internal microcracks, which deteriorates the properties. Therefore, there is an optimal grain size range in BaTiO_3_ in which the best dielectric constant values, low losses and a more stable ferroelectric response are achieved [42].

## 4. Conclusions

In this paper, the analysis of the effect of three Ba:Ti molar ratios and four sintering temperatures on the structural and electrical properties of solid solutions type BaTiO_3_ synthesized by the hydrothermal method were shown. It was demonstrated that the Ba:Ti molar ratio of 4:1 used in the synthesis allowed the formation and stability of the tetragonal ferroelectric phase of high-purity BT, which was corroborated by the XRD technique, Raman spectroscopy and HRSEM. On the other hand, it was found that, using a sintering temperature of 1300 °C, a ceramic was obtained that exhibited an apparent density value of 5.95 g/cm^3^ (99.16%), and this resulted in a structurally compact and homogeneous material. The dielectric properties of the capacitor devices measured at 100 kHz and 1 MHz over a temperature range of 30–200 °C for the sample synthesized by the hydrothermal method using a Ba:Ti ratio of 4:1 and sintered at 1300 °C showed the highest values of relative permittivity (ε′max = 4487, ε′min = 4323). The ferroelectric measurements showed typical P–E hysteresis loops at room temperature for all samples obtaining the best Pr results (3.5 µC/cm^2^), for the sample synthesized with a Ba:Ti molar ratio of 4:1 and sintered at 1325 °C at a Curie temperature of 110 °C. The HRSEM results showed that the sintered samples were characterized by a wide grain-size distribution, and a trend of increasing average grain size with increasing sintering temperature was observed 1325 °C at a Curie temperature of 110 °C.

Finally, it is concluded that the BT ceramic can be successfully synthesized by the hydrothermal method using a Ba:Ti ratio of 4:1 and a sintering temperature of 1300 °C with a heating and cooling ramp of 4 °C/min. These conditions allowed obtaining the pure ferroelectric tetragonal BT phase and the best structural and functional characteristics.

## Figures and Tables

**Table 1:** Weights of the reagents concerning the experiment analyzed.

Ba:Ti Molar Ratio	Barium Hydroxide (g)	Titanium Butoxide (g)	Sodium Hydroxide (g)	Triton X-100 (g)
1:1	2.85	5.66	2.0	0.379
2:1	5.70	5.66	2.0	0.379
4:1	9.36	5.66	2.0	0.379

**Table 2:** Structural parameters, tetragonality ratio (*c*/*a*) and crystallite size of the BT phase in the samples synthesized by hydrothermal method using a Ba:Ti = 4:1 molar ratio, and sintered in air atmosphere at 1250, 1275, 1300 and 1325 °C and a heating and cooling ramp of 4 °C/min.

Sintering Temperature (°C)	Phase	Lattice Parameters (A°)		*c*/*a*	Crystal Size (Å)
		*a*	*C*		≈45°
1250	BaTiO_3_ (JCPDS 050626)	3.995	4.023	1.0070	1025.27
1275		3.994	4.038	1.0110	1491.82
1300		3.993	4.038	1.0113	3540.87
1325		3.993	4.036	1.0108	5556.29

**Table 3:** Locations of vibration modes in Raman analysis of BT samples sintered at 1250, 1275, 1300 and 1325 °C.

Peak (cm^−1^)	Phonon	Reference
255	*A*_1_(TO)	[30]
307	(*B*_1_, *E*(TO + LO))	[12]
515	(*E*(TO), *A*_1_(TO))	[12]
718	(*E*(LO), *A*_1_(LO))	[31]

**Table 4:** Remanent polarization (Pr) values of BT capacitor devices as a function of their sintering temperature.

Sintering Temperature (°C)	Pr Estimate (µC/cm^2^)	Observations
1250	0.8	Tight curves, low material polarization
1275	1.2	Improvement in ferroelectric response
1300	2.5	Greater area on the curve and increased polarization
1325	3.5	Higher ferroelectric performance

## Data Availability

The original contributions presented in this study are included in this article. Further inquiries can be directed to the corresponding author.
